# Impact of Carbon Fluoroxide Nanoparticles on Cell Proliferation

**DOI:** 10.3390/nano11123168

**Published:** 2021-11-23

**Authors:** Alain Géloën, Gauhar Mussabek, Alexander Kharin, Tetiana Serdiuk, Sergei A. Alekseev, Vladimir Lysenko

**Affiliations:** 1 UMR Ecologie Microbienne Lyon (LEM), CNRS 5557, INRAE 1418, VetAgro Sup, Research Team “Bacterial Opportunistic Pathogens and Environment” (BPOE), Université Claude Bernard Lyon 1, 69622 Villeurbanne, France; alain.geloen@univ-lyon1.fr (A.G.); s__asha@mail.ru (A.K.); tetiana.serdiuk@gmail.com (T.S.); 2 Faculty of Physics and Technology, Al-Farabi Kazakh National University, 71, al-Farabi Ave., Almaty 050040, Kazakhstan; 3 Institute of Information and Computational Technologies, 125, Pushkin Str., Almaty 050000, Kazakhstan; 4 Institute of Engineering Physics for Biomedicine, Laboratory “Bionanophotonics”, National Research Nuclear University “MEPhI”, Kashirskoe sh. 31, 115409 Moscow, Russia; vladimir.lysenko@univ-lyon1.fr; 5 Institute of Molecular Systems Biology, Department of Biology, ETH Zurich, CH-8093 Zurich, Switzerland; 6 Chemistry Department, Taras Shevchenko National University of Kyiv, Volodymyrska Street, 64, 01601 Kyiv, Ukraine; alekseev@univ.kiev.ua; 7 Light Matter Institute, UMR-5306, Claude Bernard University of Lyon, 2 rue Victor Grignard, 69622 Villeurbanne, France

**Keywords:** nanoparticles, cellular uptake, cytotoxicity, cancer cell, xCELLigence

## Abstract

Cytotoxicity of fluorescent carbon fluoroxide (CFO) nanoparticles (NPs) was studied in a label-free manner on several cancer and non-cancer cell lines. A direct cytotoxic effect of the CFO NPs was clearly observed by a suppression of cell proliferation. The real-time measurement of cell activities allowed to quantify the impact of the uptaken NPs on cell proliferation and after washout of the NPs from the cell culture medium. The results show more toxic effects of the CFO NPs on cancer than on non-cancer cell lines. The notion of NPs biocompatibility must be related to a maximum concentration value of the NPs acceptable for a given cell type. Furthermore, the cytotoxicity effects of NPs should be studied not only during their direct exposure to cells but also after their washout from the culture medium.

## 1. Introduction

Over the past decade, nanoparticle-based techniques have been extensively explored in biomedical fields for the prevention, diagnostics, and therapy of various diseases [1,2,3]. The toxicity of nanoparticles is a key issue determining their potential for biomedical application. From this point of view, all new nanoparticles used for bioimaging and/or therapy must always be studied to evaluate their potential toxic effects on cell cultures or living organisms [4]. Systematic toxicity studies are thus essential and should be based on the characterization of physicochemical properties of nanoparticles as well as on their impact on cellular viability with the use of relevant cell models [5]. A lot of papers devoted to in vitro studies of nanoparticle’s cytotoxicity with the use of different cell lines, incubation times, and colorimetric assays were published [6,7,8].

Among the majority of nanomaterials used for biomedical applications, carbon nanoparticles deserve special attention due to their unique physical and chemical properties. Carbon nanomaterials, such as: carbon dots [9], carbon nanotubes [10], fullerenes [11], graphene [12], and nanodiamonds [13], have already been proved to be the most promising for various biomedical applications. Among the listed nanomaterials, special attention is paid to fluorescent carbon nanomaterials mainly proposed for bioimaging [14] and cancer theranostic applications [15]. In this regard, the application of carbon nanoparticles (CNPs) in clinical practice depends on their toxicity and biocompatibility. Cytotoxicity of CNPs depends on various parameters, such as: size, shape, surface charge, and surface chemistry. Numerous published studies on the cytotoxicity of CNPs remain contradictory. While some groups report on the absence of any toxic effect in different cell lines [16], other research teams observed high [17], insignificant [18] or dose-dependent cytotoxicity [19]. In addition, for the understanding of cytotoxicity mechanisms involving carbon nanoparticles, nature of cells used in experiments must be considered. Indeed, cytotoxicity of CNPs regarding cancer cells may indicate their potential application in anti-cancer therapy [20]. Such intrinsic cytotoxicity of CNPs towards cancer cells has also been reported [21,22]. 

Along with the study of pure CNPs, work is also underway to develop and study the cytotoxicity of doped carbon particles obtained by various methods [23,24]. Recently, our group reported on electrochemical synthesis of new specific kind of carbon-based nanomaterials called carbon fluoroxide nanoparticles (CFO NPs) [25]. These CFO NPs with typical sizes between 1 and 10 nm were synthesized as a sub-product during electrochemical etching of silicon carbide substrate in HF-based solutions. The structure of the CFO is akin to the structure of such irregular biopolymers, as humic acids, melanins, etc., that is why high biocompatibility of the CFO could be expected. Thermal properties of colloidal suspensions based on CFO NPs have already been studied [26], and bright fluorescent as well as ability of these particles to penetrate into cells via vapor phase allowed to use them for cell imaging [27,28]. CFO NPs were also proposed for theranostic applications with fluorescent cell imaging and ultrasonic therapy [29]. However, a detailed cytotoxicity investigation of CFO NP has not yet been carried out. It has been only reported that concentration 1 g/L of CFO NPs is toxic for 3T3-L1 fibroblast cells [29]. 

In this paper we report on a detailed study of the CFO NPs cytotoxicity to cancer cell lines versus non-cancer ones. Considering the high potential of these nanoparticles for theranostics, we focused our special attention on the investigation of the impact of CFO NPs on the proliferation of cancer cells in order to check their intrinsic therapeutic (anti-cancer) function. 

## 2. Materials and Methods

### 2.1. Preparation of Nanoparticles

CFO NPs were formed by electrochemical etching of 3C-SiC polycrystalline wafer with resistivity 1 Ohm*cm in a mixture of 50% fluoric acid and ethanol (1/1), current density 25 mA/cm^2^. After etching, the porous layer was removed from the silicon carbide wafer. CFO nanoparticles were dispersed in Dulbecco’s modified Eagle’s medium (DMEM) at a concentration of 10 mg of powder for 1 mL of DMEM and centrifuged at 20,000 *g* for 50 min to remove large size particles from the solution. The supernatant was used for further experiments. After centrifugation, the pellet represented around 90% of the initial mass of NPs powder. The real concentration of NPs to which cells were exposed was in fact 10 times lower than the initial values that are indicated on the following graphs. Chemical nature of the obtained CFO NPs was recently reported [25]. As one can read in the paper cited above, the formation of relatively small (<10 nm) CFO NPs with high content of carboxylic acid groups was achieved. 

### 2.2. Cell Cultures

All cell lines cells (from Sigma-Aldrich, Saint-Quentin-Fallavier, France) were initially grown in flasks containing Dulbecco’s modified Eagle’s medium 4.5 g/L glucose supplemented with 10% newborn calf serum 100 IU penicillin, 100 µg streptomycin, and 0.25 mg/L amphotericin B at 37 °C in a water-saturated atmosphere with 5% CO_2_, in a Heraeus incubator. Cells were trypsinised by 0.05% trypsin. Cell concentration was measured using a Sceptor pipet (Millipore). Cells were seeded at 2500 per well in a 96 wells plate—real time cell analysis (RTCA) xCELLigence plate for cell proliferation measurements. The same concentration of cells was seeded on quartz glass coverslips for photo acquisition. For cell toxicity experiments, 3T3-L1, HuH7, Panc1, HSC (HSC-2 is a human oral squamous carcinoma line), HepG2 and Hek 293 cell lines were used. 3T3-L1 and Hek 293 were non-cancer cells, while other cell lines were cancer cell lines. During the first 48 h, cells were growing under basic conditions into the cell medium inside the xCELLigence setup. Then, the nanoparticles were added to cell cultures for 24 h at concentrations: 1.5, 1, 0.5, 0.25, or 0.1 mg/mL of culture medium. After that, the NPs were washed out and replaced by fresh culture medium. Cell proliferation rates were measured over the 24 h following washing out the CFO NPs. Slopes were measured during the 12 h before addition of CFO NPs for control, during 12 h after an adaptation period of 6 h after addition of CFO NPs, and during 12 h after a period of 6 h after washout.

### 2.3. Measurements on Cell Cultures

Cell number measurements expressed in terms of a time-dependent cell index were performed using a non-destructive impedance-based method (xCELLigence, ACEA Biosciences Inc., Biotek, Colmar, France). The cells were grown on a special plate with electrodes on its bottom. The system measures electrical impedance across interdigitated microelectrodes situated at the bottom of culture wells. The measurements were conducted by applying an alternative excitation signal (20 mV control voltage amplitude) at 3 different frequencies (10, 25, and 50 kHz) through the microelectrodes in the E-plates while monitoring the voltage drop across the electrodes where the quotient voltage/current yields the impedance software shows cell index as a result of processing impedance data as a time function. The cell index was proportional to the cell number, cell surface area, and adhesion factor. For a given cell line, cell number was the main factor affecting the cell index value. The impedance measurement was a non-destructive real-time method allowing continuous measurements of cell proliferation under basic conditions, in the presence of nanoparticles and after their washing out, on the same cell population. Doubling time (the time required to double cell index value) and cell index slope on normalized curves were measured to compare the effects of increasing concentrations of the CFO NPs on the proliferation of different cell lines. Each curve was the mean of cell index measured on 8 wells.

Fluorescence of the CFO NPs was measured using Cytation 3 (Biotek, Colmar, France) equipment, a cell imaging multi-mode microplate reader combining automated digital microscopy and conventional microplate detection. Specific application of the software Gen5 (Biotek, Colmar, France) allows direct quantification of the luminescence. Photos of the cells were obtained using fluorescent microscope Zeiss axiovert 200 M (Marly le Roi, France) at ×40 magnification and excitation in blue range (wavelength of excitation band is 440–470 nm). The Emission was observed through the FITC filter (Biotek, Colmar, France), transmitting the 540–550 nm. 

### 2.4. Statistics

The measured data were expressed as: mean value ± standard error of the mean (SEM) or standard deviation (SD). Statistical analysis were performed with StatView 4.5 software (SAS Institute Inc, Évry-Grégy-sur-Yerre, France) for Windows. The data were analyzed using one-way anaylsis of variance followed by Fisher’s protected least significant difference (PLSD), post hoc test. Statistical significance was accepted at *p* < 0.05. The Spearman correlation coefficient was used to calculate a significant correlation.

## 3. Results

**Figure 1:**
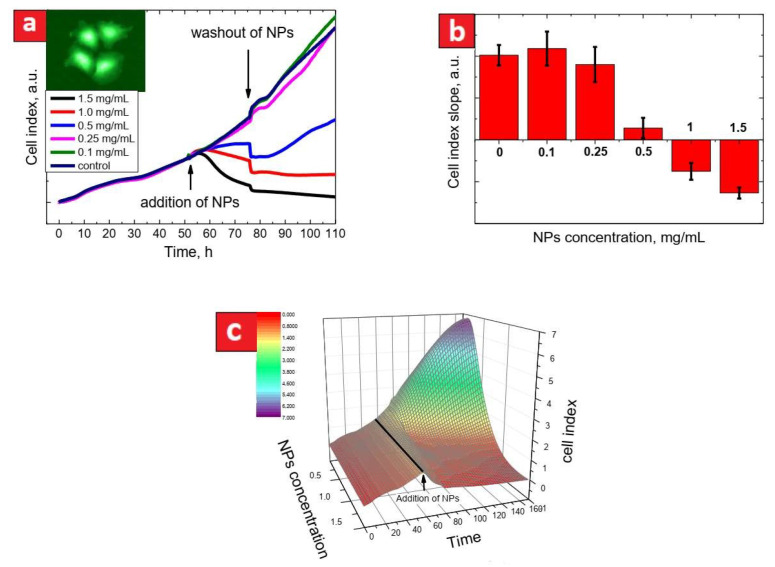
(**a**): evolution of cell index curves for HepG2 cells. Arrows show the times of addition and washing out (with a change of cell medium) of the CFO NPs. The inset shows photoluminescent image of the HepG2 cells exposed to 0.5 mg/mL of the NPs for 4 h; (**b**): the NPs toxicity for the HepG2 cells expressed in terms of cell index slopes measured in presence of CFO NPs during 50–70 h (each data column is an average of 8 curves ± SD); (**c**): toxicity surface plot (the case of HePG2 cells) represented in “time/NPs concentration/cell index” coordinates (the black line indicates the time of CFO NPs addition).

Figure 1a shows cell index evolution for the HepG2 cell line during 48 h of a control period, 24 h in the presence of CFO NPs at different concentrations and then, 24 h after washing out of the NPs. The cell index values were normalized at the time corresponding to addition of the CFO NPs. As one can see, there was no slope change during the control period, while there was a clear dose-response effect of the CFO NPs on the cell index slope. Higher CFO concentrations provoke a significant decrease in cell proliferation rates. Toxicity of the CFO NPs can be qualitatively estimated from time dependent evolution of the slope values shown in Figure 1b. For example, negative slopes indicate enhanced cytotoxicity of the CFO NPs. Indeed, the higher the CFO NPs concentration was, the more significant decrease of the cell index slope was. The highest concentration of the NPs (1.5 mg/mL) lead to the strongest reduction of cell proliferation rate for all of the tested cell lines (see Figure S1 in Supplementary Information part).

In order to represent, in another way, the real-time evolution of cell proliferation underexposure of the cells to different concentrations of the NPs, one can plot a characteristic surface in the “NPs concentration/time/cell index” coordinates. Such a representation approach can be quite useful because it may provide a more global view and complete information of the cytotoxicity effect for a given cell line. As an example, a characteristic cytotoxicity surface for the HepG2 cell line is shown in Figure 1c. As can be understood from Figure 1a,c, the concentrations below 0.25 mg/mL do not change the cell proliferation rates. In contrast, the CFO concentrations starting from 0.5 mg/mL almost completely stop the cell proliferation. 

**Figure 2:**
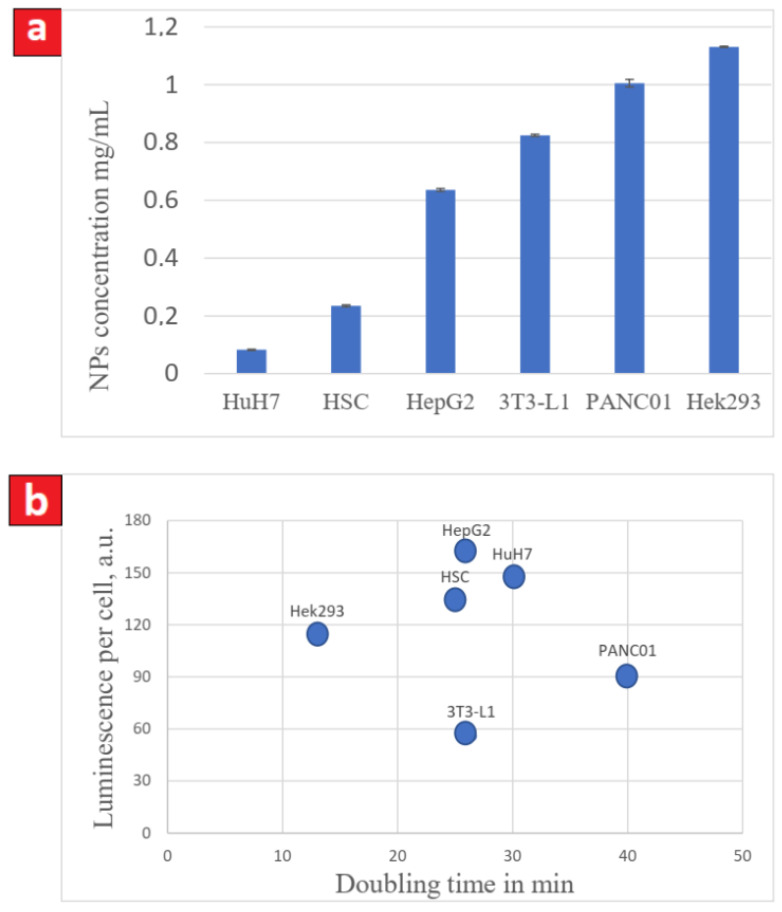
(**a**) concentrations of the CFO NPs stopping cell proliferation. The values are calculated from dose-response curves characterizing cell proliferation dynamics at different concentrations of the CFO NPs for each cell type; (**b**) correlation between the luminescence of cells (proportional to the numbers of the CFO NPs uptaken by cells) and doubling time.

To compare the cytotoxicity of the CFO NPs for different cell lines, we determined their concentrations stopping cell proliferation for each cell type (see Figure 2a). The results showed a wide range of the concentrations indicating various sensitivities of the studied cell lines to the presence of the CFO NPs. Indeed, as low as 0.2 mg/mL of CFO NPs stops cell proliferation of HuH7 while as much as 1.1 mg/mL was required to stop the proliferation of Hek293 cells.

In order to check the hypothesis assuming the enhanced CFO-induced cell toxicity for the cell lines with higher proliferation rate, we plotted in Figure 2b luminescence intensity of the cells uptaking the luminescent CFO NPs with concentrations stopping cell proliferation as a function of doubling time (the time required to double the value of the cell index and thus the cell number). Indeed, as has already been demonstrated earlier, the uptake of the NPs by cells strongly depends on their proliferation rate [30]. The results show the absence of any correlation between the cell sensitivity to the CFO NPS and cell proliferation rate (Figure 2b). 

**Figure 3:**
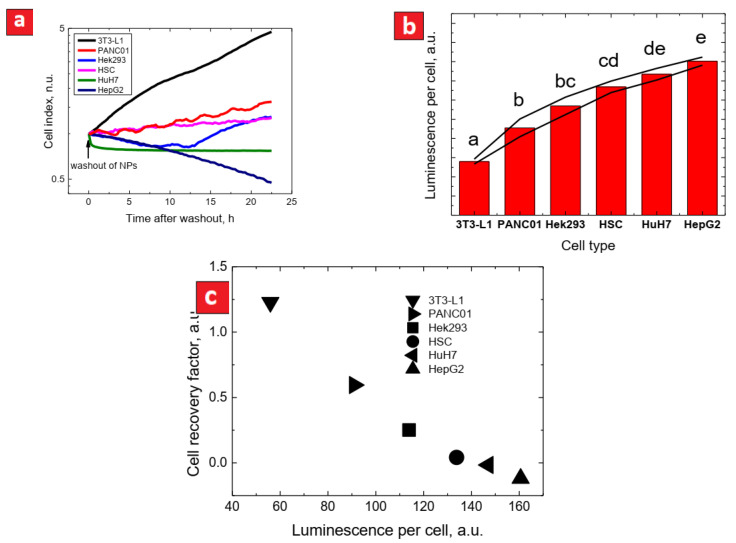
(**a**) evolution of cell indexes (normalized at the time of washout) for different cell lines after washing out 1.5 mg/mL of the CFO NPs; (**b**) luminescence per cell (proportional to the quantity of the CFO NPs uptaken by the cells) for different cell types exposed to the NPs for 4 h (each value is the result of averaging over at least 20 cells); (**c**) correlation between the luminescence per cell and cell recovery factor (CRF) after washing out (based on data from Figure 3a).

Due to the real-time measurements ensured by the xCELLigence system, it is possible to study the impact of the CFO NPs after their washing out from the cell culture medium. In that case, only the definitely uptaken CFO NPs can still impact the cell proliferation rates. The cell index evolution after exposure of different cell lines to 1.5 mg/mL of the CFO NPs for 24 h with consequent washing out of the NPs, is shown in Figure 3a. To compare cell proliferation rates after the washout for different cell lines, we calculated a cell recovery factor (CRF). The CRF is a relative recovery of the cell index slope after washout, which is defined as follows:

$$CRF\;\left(cell\;recovery\;factor\right)=\frac{slope\left(1.5\;mg/mL\;after\;washout\right)}{slope\left(initial\right)}$$

All the cell index values were normalized at the washout time. As one can conclude from Figure 3a, there was a striking difference after the washing out between the 3T3-L1 and the other cancer cell lines. Since the differences in cytotoxicity after washout can be expected to be also dependent on different capacities of cells to uptake the luminescent NPs, we estimated relative amount of the NPs uptaken by the different cell lines in terms of luminescence intensity per cell (see Figure 3b). The results of such a comparison were based on measurements of at least 20 cells. It can be seen that the 3T3-L1 cells uptake much less NPs than the other cell lines. The 3T3-L1 cells did not uptake many NPs, and, as a result, their proliferation restarted after washing out the NPs. PANC01, Hek293, and HSC cancer cells uptake more NPs and their proliferations restarted at much lower rates. HepG2 and HuH7 cancer cells uptake much more NPs, and their proliferations did not restart at all. Thus, one can state a significant correlation between the NPs uptake estimated by the cell luminescence and the CRF values, as summarized in Figure 3c.

## 4. Discussion

Development of new analytical tools allowing a non-invasive real-time measurement of cell proliferation brings new opportunities for testing cytotoxicity effects. In the context of cancer treatment, inhibition of cancer cell proliferation is a major target. In our experimental protocol, the first period of 48 h of cell proliferation without any treatment allows a qualitative control of the studied cell lines. Indeed, the in-time shape of a cell index curve is an important characteristic of a cell type evolution. Knowing the quantity of seeded cells, one may expect to obtain a very reproducible cell index after 48 h, which is an appropriative control parameter for each cell line (Figure 1a). 

The real-time measurement of cell proliferation gives an opportunity to measure direct effects of NPs on various cell lines. This is a significant advantage compared to the endpoint measurement such as, for example, MTT approach or other similar methods. The direct impacts of NPs on different cell lines can be visualized and quantified by comparison of the slopes of cell proliferation curves either with a control non-treated line or (even better) with the same cell population before treatment (Figure 1b). Furthermore, most of in vitro cytotoxicity studies do not pay enough attention to the great differences between in vivo and in vitro conditions. For example, one of the main differences is related to impossibility to keep locally a high concentration of NPs during in vivo experiments. Indeed, after injection, NPs are often rapidly bound to surrounding bio-compounds and either uptaken by cells or eliminated from the body. Thus, the best model to study cytotoxicity effects of NPs is a short time exposure of cells to high concentrations of NPs with a consequent significant decrease of their concentration or even with their total disappearance from the environment. Washing out of the NPs from the culture medium gives an opportunity to study cytotoxic effects of intracellular NPs. As can be seen in Figure 1 and corresponding Supplementary Figure S2, the cytotoxicity behavior of intracellular NPs is different from that observed when they are also present in the cell culture medium. One way to quantify the effect of intracellular NPs is to compare the slope of cell proliferation after washing out of NPs at the highest concentration (1.5 mg/mL) with the initial slope of the cell proliferation curves. This is what we call the Cell Recovery Factor (CRF). The main advantage of the CRF is to make possible a comparison between different cell lines having different initial cell proliferation rates. It is worth noting that the cytotoxicity of the CFO NPs after their washing out is very different for non-cancer cells (3T3-L1, Hek293) compared to the cancer cell lines (HepG2, Panc01, HuH7, HSC). Indeed, the cell proliferation restarted after the NPs washing out for the case of non-cancer cells, contrary to the cancer cell lines after their exposure to the highest concentration values of NPs. Finally, the global 3D representation of cell proliferative curves allows the analysis at a glance of the results (Figure 1c).

The intracellular concentration of the CFO NPs stopping cell proliferation was found to be very variable for different cell lines (Figure 2a). It does not correlate to cancer or non-cancer nature of the studied cells lines. Indeed, if as little as 0.1 mg/mL was enough to stop HuH7 cell proliferation, at least 0.8 mg/mL and 1 mg/mL are required to stop proliferation, respectively, of 3T3-L1 and Panc01 cell lines. In addition, no correlation was found between the concentrations of the NPs uptaken by the cells and the slope of cell proliferation, (i.e., the proliferation rate). The differences in the concentrations stopping cell proliferation vary from 1 to 10, and it strongly depends on the type of the studied cell lines. This important observation suggests that the biocompatibility of the NPs is limited to the highest concentration of NPs, showing no toxic effect for a given cell line. In other words, if the same concentration value of NPs can be acceptable for a given cell line, it can appear as toxic for another one.

Another important issue of the present study concerns the fact that the biocompatibility should be rather referenced to the intracellular concentration of the NPs instead of the extracellular one [31]. Indeed, there was a perfect correlation between the CRF values and the luminescence per cell defined by the uptaken luminescent CFO NPs (Figure 3c), suggesting that the effects of the NPs on cell proliferation strongly depends on their intracellular concentration. Although it seems to be obvious, it is not a trivial observation. Indeed, the NPs could, for example, bind plasma membrane and exert deleterious effects without entering inside cells. We also found no correlation between the uptaken luminescent NPs (expressed in terms of luminescence per cell) and the doubling time (Figure 2b). This result must be considered with caution. Indeed, the intracellular concentration of NPs has been measured after only 4 h of their exposure to cells, while cell division takes hours. Thus far, we have no data on the kinetic of CFO uptake by cells: is it linear? is there a saturation threshold? Assuming that the uptake of the NPs is linear over time and that the saturation threshold is the NPs concentration at which cells show alterations/ex stop division, these results indeed suggest that there is no relation between the rate of cell division and the NPs uptake during incubation. That implies that the effects of NPs are dependent on the cancerous nature of cells but not on their increased division rate. One explanation may lie in the fact that the CFO NPs are predominantly accumulated inside the nuclei of cells (Figure 1a) as reported before [5]. Although we have no direct evidence of the nature of the interaction of the CFO NPs and chromatin, since the cancer cells are characterized by an increased cell proliferation rate than the non-cancer ones, it may be hypothesized that they are more sensitive to the action of the CFO NPs. That may not be true for any type of cancer cell line. The fact that we observed a close relationship between the NPs uptaken by cells and the CRF suggests that the effects of the NPs after a short exposure time are rather privileged. In the vast majority of the studies on NPs only the consequences of direct exposure of cells are examined. The present study shows that a short-time exposure of cells to the NPs and the analysis of their effects after washing out is relevant and most likely closer to physiological conditions than direct exposure.

## 5. Conclusions

In summary, NPs cytotoxicity must be studied in two cases: (i) direct effects of the NPs on cell lines after their introduction to the cell medium and (ii) the effects appearing after washing out of the NPs from the medium. The CFO NPs have been shown to be less toxic for non-cancer cells (3T3-L1) than for the cancer cell lines (HSC, HuH7, Panc01, HepG2). No correlation was found between cell proliferation rate and level of the NPs uptake. In particular, it is not because the cancer cells proliferate more, they uptake more NPs. The same NPs shows different cytotoxicity towards different cell lines. It means that biocompatibility notion alone is meaningless, it must be mentioned in reference to a concentration value of NPs for a given cell type. Thus, Paracelse’s citation “Poison is in everything, and no thing is without poison. The dosage makes it either a poison or a remedy” can be also applied to the case of NPs. 

## Supplementary Materials

The following are available online at https://www.mdpi.com/article/10.3390/nano11123168/s1, Figure S1: Dose-response of cell index of different cell lines in presence of increasing concentrations of CFO NPs; Figure S2: The toxicity surfaces of the CFO NPs (based on Figure S1).

## References

[1] Pratiwi F., Kuo C.W., Chen B.-C., Chen P. (2019). Recent advances in the use of fluorescent nanoparticles for bioimaging. Nanomedicine.

[2] Fu D., Liu D., Zhang L., Sun L. (2020). Self-assembled fluorescent tripeptide nanoparticles for bioimaging and drug delivery applications. Chin. Chem. Lett..

[3] Molkenova A., Toleshova A., Song S.-J., Kang M.S., Abduraimova A., Han D.-W., Atabaev T.S. (2020). Rapid synthesis of nontoxic and photostable carbon nanoparticles for bioimaging applications. Mater. Lett..

[4] Love S.A., Maurer-Jones M.A., Thompson J.W., Lin Y.-S., Haynes C.L. (2012). Assessing nanoparticle toxicity. Annu. Rev. Anal. Chem..

[5] Filali S., Geloën A., Lysenko V., Pirot F., Miossec P. (2018). Live-stream characterization of cadmium-induced cell death using visible CdTe-QDs. Sci. Rep..

[6] Lewinski N., Colvin V., Drezek R. (2008). Cytotoxicity of nanoparticles. Small.

[7] Patil R.M., Thorat N.D., Shete P.B., Bedge P.A., Gavde S., Joshi M.G., Tofail S.A., Bohara R.A. (2018). Comprehensive cytotoxicity studies of superparamagnetic iron oxide nanoparticles. Biochem. Biophys. Rep..

[8] Barbasz A., Oćwieja M., Piergies N., Duraczyńska D., Nowak A. (2021). Antioxidant-modulated cytotoxicity of silver nanoparticles. J. Appl. Toxicol..

[9] Devi P., Saini S., Kim K.-H. (2019). The advanced role of carbon quantum dots in nanomedical applications. Biosens. Bioelectron..

[10] Merum S., Veluru J.B., Seeram R. (2017). Functionalized carbon nanotubes in bio-world: Applications, limitations and future directions. Mater. Sci. Eng. B.

[11] Castro E., Garcia A.H., Zavala G., Echegoyen L. (2017). Fullerenes in biology and medicine. J. Mater. Chem. B.

[12] Nie C., Ma L., Li S., Fan X., Yang Y., Cheng C., Zhao W., Zhao C. (2019). Recent progresses in graphene-based bio-functional nanostructures for advanced biological and cellular interfaces. Nano Today.

[13] Zhang K., Zhao Q., Qin S., Fu Y., Liu R., Zhi J., Shan C. (2019). Nanodiamonds conjugated upconversion nanoparticles for bio-imaging and drug delivery. J. Colloid Interface Sci..

[14] Chandra S., Das P., Bag S., Laha D., Pramanik P. (2011). Synthesis, functionalization and bioimaging applications of highly fluorescent carbon nanoparticles. Nanoscale.

[15] Kumar V., Toffoli G., Rizzolio F. (2013). Fluorescent carbon nanoparticles in medicine for cancer therapy. ACS Med. Chem. Lett..

[16] Chen Y.-Y., Jiang W.-P., Chen H.-L., Huang H.-C., Huang G.-J., Chiang H.-M., Chang C.-C., Huang C.-L., Juang T.-Y. (2021). Cytotoxicity and cell imaging of six types of carbon nanodots prepared through carbonization and hydrothermal processing of natural plant materials. RSC Adv..

[17] Li D., Na X., Wang H., Xie Y., Cong S., Song Y., Xu X., Zhu B.-W., Tan M. (2018). Fluorescent carbon dots derived from Maillard reaction products: Their properties, biodistribution, cytotoxicity and antioxidant activity. J. Agric. Food Chem..

[18] Singh S., Singh D., Singh S.P., Pandey A.K. (2019). Candle soot derived carbon nanoparticles: Assessment of physico-chemical properties, cytotoxicity and genotoxicity. Chemosphere.

[19] Kumar P., Meena R., Paulraj R., Chanchal A., Verma A., Bohidar H. (2012). Fluorescence behavior of non-functionalized carbon nanoparticles and their in vitro applications in imaging and cytotoxic analysis of cancer cells. Colloids Surf. B Biointerfaces.

[20] Kumar D., Kumar Sharma P. (2018). Nanoparticulate system for cancer therapy: An updated review. Int. J. Nanomater. Nanotechnol. Nanomed..

[21] Khashan K.S., Abdulameer F.A., Jabir M.S., Hadi A.A., Sulaiman G.M. (2020). Anticancer activity and toxicity of carbon nanoparticles produced by pulsed laser ablation of graphite in water. Adv. Nat. Sci. Nanosci. Nanotechnol..

[22] Boobalan T., Sethupathi M., Sengottuvelan N., Kumar P., Balaji P., Gulyás B.Z., Padmanabhan P., Selvan S.T., Arun A. (2020). Mushroom-derived carbon dots for toxic metal ion detection and as antibacterial and anticancer agents. ACS Appl. Nano Mater..

[23] Barkhudarov E.M., Kossyi I.A., Anpilov A.M., Ivashkin P.I., Artem’Ev K.V., Moryakov I.V., Misakyan M.A., Christofi N., Burmistrov D.E., Smirnova V.V. (2020). New nanostructured carbon coating inhibits bacterial growth, but does not influence on animal cells. Nanomaterials.

[24] Guo J., Ye S., Li H., Song J., Qu J. (2020). Novel fluorescence probe based on bright emitted carbon dots for ClO– detection in real water samples and living cells. Spectrochim. Acta Part A Mol. Biomol. Spectrosc..

[25] Alekseev S., Korytko D., Iazykov M., Khainakov S., Lysenko V. (2015). Electrochemical synthesis of carbon fluorooxide nanoparticles from 3C-SiC substrates. J. Phys. Chem. C.

[26] Dubyk K., Isaiev M., Alekseev S., Burbelo R., Lysenko V. (2019). Thermal conductivity of nanofluids formed by carbon flurooxide mesoparticles. SN Appl. Sci..

[27] Serdiuk T., Alekseev S., Lysenko V., Skryshevsky V., Géloën A. (2014). Trypsinization-dependent cell labeling with fluorescent nanoparticles. Nanoscale Res. Lett..

[28] Serdiuk T., Lysenko V., Skryshevsky V.A., Géloën A. (2012). Vapor phase mediated cellular uptake of sub-5 nm nanoparticles. Nanoscale Res. Lett..

[29] Kharin A., Syshchyk O., Géloën A., Alekseev S., Rogov A., Lysenko V., Timoshenko V. (2015). Carbon fluoroxide nanoparticles as fluorescent labels and sonosensitizers for theranostic applications. Sci. Technol. Adv. Mater..

[30] Serdiuk T., Lysenko V., Mognetti B., Skryshevsky V., Géloën A. (2013). Impact of cell division on intracellular uptake and nuclear targeting with fluorescent SiC-based nanoparticles. J. Biophotonics.

[31] Géloën A., Isaieva K., Isaiev M., Levinson O., Berger E., Lysenko V. (2021). Intracellular detection and localization of nanoparticles by refractive index measurement. Sensors.

