# Supplementary material for: Impact of Carbon Fluoroxide Nanoparticles on Cell Proliferation
Source: Nanomaterials (Basel). 2021 Nov 23;11(12):3168. doi: 10.3390/nano11123168 (PMC8708860; doi:10.3390/nano11123168)
Supplement: Supplementary file 1 [file nanomaterials-11-03168-s001.zip › nanomaterials-1427283-supplementary.pdf]

Supplementary Materials

Impact of carbon fluoroxide nanoparticles on proliferation of cancer cells
Alain Géloën, Gauhar Mussabek*, Alexander Kharin, Tetiana Serdiuk, Sergei A. Alekseev and Vladimir Lysenko

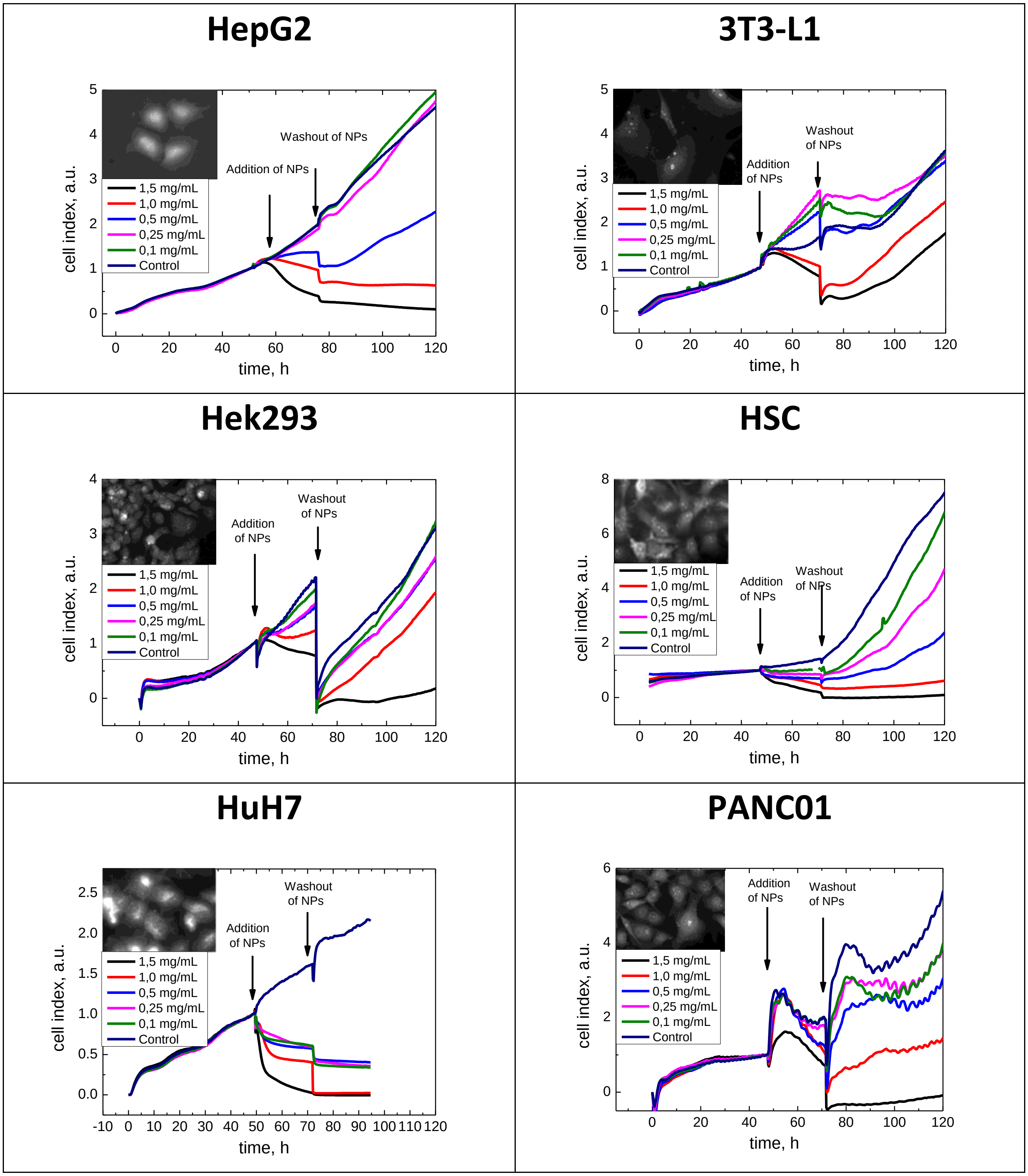

Figure S1. Dose-response of cell index of different cell lines in presence of increasing concentrations of CFO NPs.

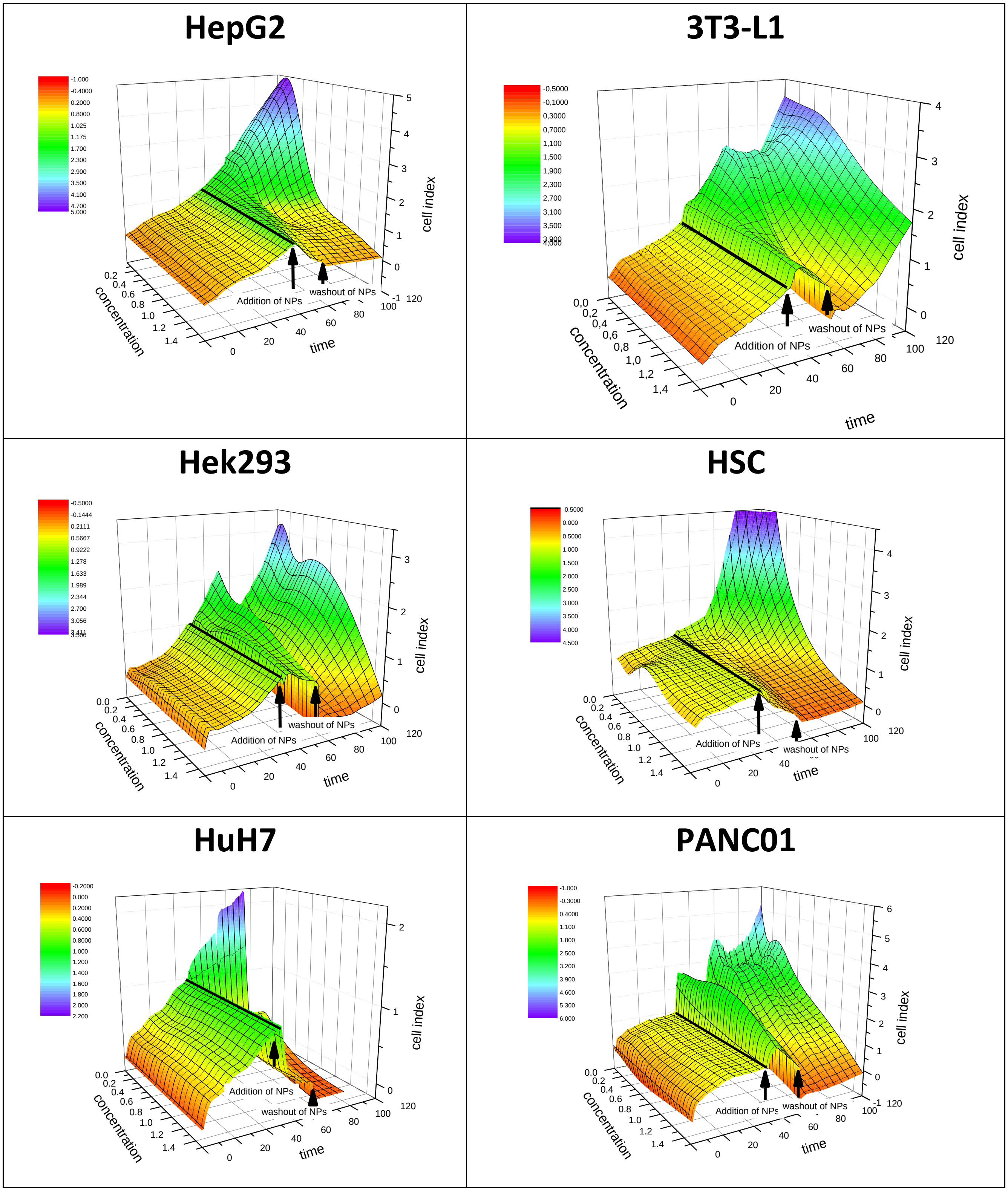

Figure S2. The toxicity surfaces of the CFO NPs (based on Figure S1).
